# Combination of Systemic Inflammatory Biomarkers in Assessment of Chronic Obstructive Pulmonary Disease: Diagnostic Performance and Identification of Networks and Clusters

**DOI:** 10.3390/diagnostics10121029

**Published:** 2020-11-30

**Authors:** Iva Hlapčić, Daniela Belamarić, Martina Bosnar, Domagoj Kifer, Andrea Vukić Dugac, Lada Rumora

**Affiliations:** 1 Department of Medical Biochemistry and Haematology, Faculty of Pharmacy and Biochemistry, University of Zagreb, 10000 Zagreb, Croatia; iva.hlapcic@pharma.unizg.hr; 2 Fidelta Ltd., 10000 Zagreb, Croatia; daniela.belamaric@glpg.com (D.B.); Martina.Bosnar@glpg.com (M.B.); 3 Department of Biophysics, Faculty of Pharmacy and Biochemistry, University of Zagreb, 10000 Zagreb, Croatia; domagoj.kifer@pharma.unizg.hr; 4 Clinical Department for Lung Diseases Jordanovac, University Hospital Centre Zagreb, 10000 Zagreb, Croatia; avukic@kbc-zagreb.hr; 5 School of Medicine, University of Zagreb, 10000 Zagreb, Croatia

**Keywords:** chronic obstructive pulmonary disease, cytokines, systemic inflammation, clusters, adenosine triphosphate, heat shock protein 70

## Abstract

Interleukin (IL)-1α, IL-1β, IL-6, IL-8 and tumor necrosis factor (TNF)α contribute to inflammation in chronic obstructive pulmonary disease (COPD). We wanted to investigate their interrelations and association with disease severity, as well as to combine them with other inflammation-associated biomarkers and evaluate their predictive value and potential in identifying various patterns of systemic inflammation. One hundred and nine patients with stable COPD and 95 age- and sex-matched controls were enrolled in the study. Cytokines’ concentrations were determined in plasma samples by antibody-based multiplex immunosorbent assay kits. Investigated cytokines were increased in COPD patients but were not associated with disease or symptoms severity. IL-1β, IL-6 and TNFα showed the best discriminative values regarding ongoing inflammation in COPD. Inflammatory patterns were observed in COPD patients when cytokines, C-reactive protein (CRP), fibrinogen (Fbg), extracellular adenosine triphosphate (eATP), extracellular heat shock protein 70 (eHsp70) and clinical data were included in cluster analysis. IL-1β, eATP and eHsp70 combined correctly classified 91% of cases. Therefore, due to the heterogeneity of COPD, its assessment could be improved by combination of biomarkers. Models including IL-1β, eATP and eHsp70 might identify COPD patients, while IL-1β, IL-6 and TNFα combined with CRP, Fbg, eATP and eHsp70 might be informative regarding various COPD clinical subgroups.

## 1. Introduction

Cytokines are small proteins (5–30 kDa) with a short half-life and they are usually circulating in body fluids in picomolar concentrations. While being produced by a variety of cells, their main role is regulation of the immune system, so they or their receptors are often being recognized as targets for potential therapeutic interventions in many different diseases [1]. Cytokines are not disease-specific biomarkers, yet they are considered to be surrogate biomarkers for inflammation in chronic obstructive pulmonary disease (COPD), as it seems they have an important role in COPD-associated inflammatory responses [2]. COPD is a complex, heterogeneous disease at the genetic (e.g., alpha-1 antitrypsin deficiency), cellular and molecular levels, and its manifestations are both pulmonary and extrapulmonary [3]. Currently, it is the fourth leading cause of death in the world, and it represents an important public health challenge as its global prevalence of 11.7% is expected to rise for many years to come [4]. Although COPD is characterized by respiratory symptoms and airflow limitation, systemic inflammation may be developed in some patients and it contributes to the progression of the disease and development of comorbidities that might have an impact on morbidity and mortality [5,6,7,8,9]. Various clinical studies reported elevated levels of inflammatory cytokines in respiratory tract and/or peripheral blood of COPD patients in comparison to healthy controls [2,7,10,11,12]. Our study focused on several cytokines as it follows: interleukin (IL)-1α, IL-1β, IL-6, IL-8 and tumor necrosis factor (TNF)α.

IL-1 is mainly produced by the airway epithelium and macrophages, and it is released along with IL-6, IL-8 and TNFα. It causes neutrophilia, macrophage activation and responses by T cells [13]. Both pro-IL-1α and its mature IL-1α form are biologically active [14]. Contrary to this, pro-IL-1β has to be cleaved to be biologically active, mostly by caspase-1 through nucleotide-binding domain (NOD)-like receptor protein (NLRP)3 inflammasome activation [15]. IL-6 is a pro-inflammatory cytokine synthesized by the airway epithelium, macrophages, and other cells at the site of inflammation in response to environmental stressful stimuli such as smoking, and it is participating in the activation, proliferation and differentiation of T cells [6,16,17]. IL-8 is a multifunctional chemokine involved in inflammatory processes including neutrophil infiltration and chemotaxis [16,18]. It is secreted from macrophages, T cells, airway epithelium and neutrophils [19]. TNFα is produced by T cells, mast cells, and cells of airway epithelium. Its main functions are control of cellular migration and stimulation of secretion of other cytokines [20].

There are inconsistent observations regarding the association of cytokines with COPD severity, prognostic value and cytokine-targeted therapeutic approach. In addition, due to the disease complexity and different underlying mechanisms, clinical manifestations of COPD are presented differently. Therefore, instead of one, a group of biomarkers might better represent a specific COPD phenotype. In line with this, it was shown that persistent systemic inflammation is present in some of COPD patients and accompanied with an increase in CRP, fibrinogen (Fbg), white blood cells (WBC) and inflammatory cytokines [9]. Agusti et al. investigated six inflammatory parameters (CRP, Fbg, WBC, IL-6, IL-8 and TNFα) which form “inflammome” and showed that 70% of COPD patients had some of the components of systemic inflammation. Among them, in 16% of COPD patients inflammation was persistent, and associated with mortality and exacerbations [21]. From our previous studies, we observed that our COPD cohort might also show characteristics of systemic inflammation because of increased concentrations of CRP and Fbg [22] that are being common inflammatory parameters as well as extracellular adenosine triphosphate (eATP) [23] and extracellular heat shock protein 70 (eHsp70) [24] which act like damage-associated molecular patterns (DAMPs). In addition, a previous investigation showed that there were significant associations between the aforementioned parameters with lung function and disease severity, as well as symptoms severity and history of exacerbations, and different multicomponent clinical parameters used for the assessment of dyspnoea, exacerbations and lung impairment. These parameters have not been studied together before, and we wanted to evaluate their combined performances. First, our aim was to determine concentrations of cytokines IL-1α, IL-1β, IL-6, IL-8 and TNFα in COPD patients in comparison to healthy subjects and to investigate their association with disease and symptoms severity. As cytokines exhibit pleiotropy and redundancy, we also wanted to assess relations between them in healthy non-smokers, healthy smokers and COPD patients. We hypothesized that the combination of common inflammatory biomarkers (CRP and Fbg), DAMPs (eATP and eHsp70) and cytokines might ameliorate the understanding of relations between different inflammatory parameters and help to identify some potential COPD subgroups regarding systemic inflammation. Finally, we wanted to suggest a model of combined parameters for recognizing COPD patients based on predictive value.

## 2. Materials and Methods

### 2.1. Participants

The current cross-sectional case-control study included 109 patients with stable COPD and 95 healthy individuals. For the additional analyses that involved eATP, one COPD patient was excluded because the plasma sample for the determination of eATP could not be obtained. For the determination of eATP and eHsp70, all individuals from the study (137 COPD patients and 95 controls) were recruited during 2017 and 2018 at the Clinical Department for Lung Diseases Jordanovac, University Hospital Centre Zagreb (Zagreb, Croatia) according to the predefined inclusion and exclusion criteria, while additional recruitment for the investigation of common inflammatory biomarkers and cytokines was performed during 2019 (109 COPD patients and 95 controls). During the second recruitment, not all participants were suitable to be included in the study because some of them died (*n* = 10), while others did not match inclusion criteria (lung transplantation, *n* = 5; acute exacerbations, *n* = 4) or could not be reached (*n* = 9). All participants agreed to take a part in the study as volunteers and confirmed it by signing an informed consent. The study was approved by the Ethics Committee of University of Hospital Centre Zagreb and Ethics Committee for Experimentation of Faculty of Pharmacy and Biochemistry, University of Zagreb (Zagreb, Croatia) (Approval Protocol Numbers: 02/21/JG on 29 August 2014 and 251-62-03-14-78 on 10 September 2014, respectively). Pulmonology specialists confirmed diagnosis of COPD after symptoms evaluation and spirometry measurements according to the guidelines by the Global Initiative for Chronic Obstructive Pulmonary Disease (GOLD) [4]. All patients were in the stable phase of the disease with no exacerbations in the last three months since the recruitment, no changes in therapy and no symptoms of infection in lower respiratory tract. On the other hand, healthy individuals were included in the study based on anamnestic data and spirometry results that were among normal values. They were age- and gender-matched to the COPD patients. Exclusion criteria were same for all participants and they included as follows: age under 40, lung diseases other than COPD (except COPD for COPD patients), systemic inflammatory diseases, acute infections, diabetes with severe complications, severe liver diseases, severe kidney insufficiency, malignant diseases, transplantations, and other specific or non-specific acute inflammations. In addition, smoking data was obtained from all participants. COPD patients were divided in GOLD 2-4 stages according to the level of airflow limitation, as suggested by GOLD guidelines [4]. Besides forced expiratory volume in one second (FEV_1_)-based disease severity, COPD patients were divided in GOLD A-D groups based on the assessment of symptoms severity and history of exacerbations. Evaluation of the symptoms and health-related quality of life was assessed by COPD Assessment Test (CAT), modified Medical Research Council (mMRC) Dyspnoea Scale as well as St George Respiratory Questionnaire for COPD patients (SGRQ-C). Additionally, data about previous exacerbations were obtained from the COPD patients. Finally, the Charlson comorbidity index was matched to every COPD patient, so that the multicomponent parameter CODEx could be established. CODEx stands for comorbidities (Charlson index), airflow obstruction, dyspnoea, and previous exacerbations [25].

### 2.2. Evaluation of Lung Function

Diagnosis of airflow limitation was established by spirometry when FEV_1_ and forced vital capacity (FVC) ratio was <0.70. Measurements were performed by trained technicians at the Clinical Department for Lung Diseases Jordanovac, University Hospital Centre Zagreb. Moreover, the pulmonary diffusion capacity for carbon monoxide (DLCO) was measured for the assessment of lung function in COPD patients. Both procedures were performed as already described in detail in [23].

### 2.3. Blood Sampling and Cytokine Determination

Peripheral venous blood was collected from 7 a.m. to 9 a.m. by venepuncture of a large antecubital vein after overnight fasting. Tubes with K_3_-ethylenediaminetetraacetic acid (K_3_ EDTA) anticoagulant (Greiner Bio-One, Kremsmünster, Austria) were used for the blood collection. Afterwards, tubes were mixed by an inversion 8×, and centrifuged immediately, as recommended by the Clinical and Laboratory Standards Institute (CLSI) guidelines [26]. Obtained EDTA plasma samples were stored at −80 °C until the analysis. Concentration of IL-1α in plasma was determined by Platinum Procarta Plex Kit (Thermo Fischer Scientific, Waltham, MA, USA), while levels of IL-1β, IL-6, IL-8 and TNFα were determined by Procarta Plex High Sensitivity Luminex kit (Thermo Fischer Scientific), according to manufacturer’s recommendations. Antibody-coated magnetic beads were transferred to wells of a 96-well plate and washed. Afterwards, 25 µL of assay buffer was added to wells followed by addition of 25 µL of samples or standards. For IL-1α, determination plate was incubated for 120 min at room temperature (RT) with shaking, while for determination of the other cytokines, plates were incubated for 30 min at RT followed by an overnight incubation at 4 °C. At the end of incubation, plates were washed and 25 µL of detection antibodies were added to wells. Plates were then incubated for 30 min at RT, with shaking. After the washing step, 50 µL of streptavidin-phycoerythrin conjugate was added to wells and plates were incubated for 30 min at RT with shaking. At the end of incubation, plates for IL-1α determination were washed, 120 µL of reading buffer was added to wells and samples were analyzed by use of Luminex 200 instrument (Luminex Corporation, Austin, TX, USA). On the other hand, for IL-1β, IL-6, IL-8 and TNFα determination, 50 µL of amplification reagent 1 was added to wells. After 30 min incubation, 50 µL of amplification reagent 2 was added to wells and incubation continued for additional 30 min. Finally, plates were washed, beads were resuspended in 120 µL of reading buffer, and samples were analyzed by a Luminex 200 instrument. Cytokines concentrations were determined by interpolation from a standard curve using the xPONENT software package (Luminex Corporation).

### 2.4. Statistics

Normality of all data was tested by Kolmogorov–Smirnov test, and since all data failed a normality test, results were shown as median with interquartile range (IQR). Only age was shown as median with minimum and maximum, while gender was shown in absolute numbers. Non-parametric Mann–Whitney test and Kruskal–Wallis test were used for the analyses of differences between the groups of interest. Gender was tested by Chi-squared test. Univariate and multivariate logistic regression analyses were used to investigate COPD-inflammation contributing factors, and odds ratio (OR) with 95% confidence interval (CI) were obtained. Variables were added in the binary logistic regression analysis as continuous variables. Described analysis were performed in MedCalc statistical software version 17.9.2. (MedCalc Software, Ostend, Belgium).

Network analysis was used for the assessment of relations between investigated parameters. Values of the 95th percentile of each parameter in healthy non-smokers were considered as the criteria for the evaluation of the patterns between the parameters in healthy non-smokers, healthy smokers and COPD patients. Differences in the number of patients with abnormal levels between the groups were analyzed using Fisher’s exact tests. Prior hierarchical clustering, variables were transformed to standard normal distribution by inverse transformation of ranks to normality (R package “Gen ABEL”) [27]. Distance between the subjects was calculated using Euclidian method, and group of subjects were merged by complete linkage method. Optimal number of clusters was determined combining 30 indices apply NbClust function (R package “NbClust”) [28]. Variables used for clustering and reference variables presented next to cluster were compared using a Kruskal–Wallis test or Fisher’s exact test, depending on the data type. Network and clustering analyses were performed in R programming software (R Core Team) [29]. For all analyses, the false discovery rate was controlled using the Benjamini–Hohcberg method at significance level of 0.05.

## 3. Results

### 3.1. Basic Characteristics and Cytokines’ Concentrations of All Participants

One hundred and nine patients with stable COPD were compared to age- and gender-matched healthy subjects (total healthy participants and only healthy non-smokers). COPD patients showed to have declined lung function in comparison to controls assessed by spirometry parameters. All investigated cytokines were elevated in peripheral circulation of COPD patients when compared to healthy individuals (Table 1). As smoking data were obtained from all participants, it was shown that only TNFα was increased in healthy smokers in comparison to healthy non-smokers. In addition, concentrations of TNFα and IL-6 were increased in both COPD former smokers and COPD smokers when they were compared to healthy controls regarding their smoking status as well as to COPD non-smokers. On the other hand, IL-1α was increased only in COPD smokers in comparison to both non-smoking controls and smoking controls, while IL-1β showed to be increased in each of COPD groups regarding smoking status when compared to healthy non-smokers and healthy smokers (see Supplementary Table S1).

### 3.2. Association of Cytokines’ Concentrations with the Severity of Airflow Limitation and Symptoms Severity

All the cytokines were investigated regarding the severity of COPD based on GOLD guidelines (Table 2). None of the cytokines was associated with the severity of airflow obstruction or the symptoms severity and history of exacerbations, since the concentrations were only elevated in each of GOLD 2-4 stages and GOLD A-D groups when being compared to healthy subjects but did not differ between GOLD 2-4 or GOLD A-D. IL-8 was the only cytokine whose level did not show significant difference between healthy subjects and COPD patients with moderate COPD in GOLD 2 stage, and there was no change of IL-8 concentration in either of GOLD A-D groups. As well as in combined ABCD assessment, we have compared cytokines’ concentrations in COPD frequent exacerbators and non-frequent exacerbators, but no statistically significant difference was found (data not shown). In addition, cytokines’ concentrations were similar in men and women (in both healthy and COPD groups). Regarding comorbidities (cardiovascular diseases or metabolic diseases), we also found no statistically significant difference in circulating cytokines’ levels.

### 3.3. Cytokines’ Interrelations

**Figure 1:**
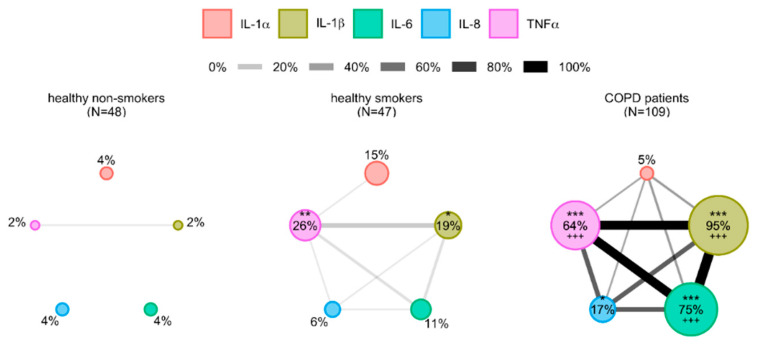
Network layout of cytokines determined in healthy non-smokers, healthy smokers and COPD patients. Cytokines are shown as different nodes of the network whose size is in proportion with the prevalence of their abnormal values defined by 95th percentile of healthy non-smokers. Two nodes are linked when more than 1% of participants in the network share abnormal values of these two parameters, and width of a line is proportional to that proportion. IL-1α–interleukin-1alpha; IL-1β–interleukin-1beta; IL-6–interleukin-6; IL-8–interleukin-8; TNFα–tumor necrosis factor alpha. * *p* < 0.05, ** *p* < 0.01, *** *p* < 0.001 in comparison to healthy non-smokers, +++ *p* < 0.001 in comparison to healthy smokers.

Network analysis was performed for the assessment of relations between investigated cytokines. Every cytokine was presented by an individual node, and its size was proportional to the percentage of defined abnormal values, as described (see Supplementary Table S2). Links between the nodes were present when at least 1% of the participants shared abnormal values for linked parameters. Moreover, the width of the link presented the percentage of the participants sharing abnormal values (Figure 1). There were no significant cytokine-based interrelations in healthy non-smokers as the nodes were small and the links between them were rare. Interestingly, healthy smokers showed to have larger nodes of IL-1β (*p* < 0.05) and TNFα (*p* < 0.01), and there were more linking nodes in comparison to healthy non-smokers. The cytokine network is even more developed in COPD patients with increased nodes of IL-1β, IL-6 and TNFα (*p* < 0.001 in comparison to both healthy non-smokers and healthy smokers for all three parameters) as well as IL-8 (*p* < 0.05 in comparison to healthy non-smokers).

### 3.4. The Potential of Cytokines in Identifying COPD Patients

To determine the potential of cytokines regarding identifying COPD patients, univariate logistic regression analysis was performed for cytokines whose concentrations were determined in plasma of all participants from the study. IL-1α had no statistically significant predictive performances, while IL-8 showed to have the lowest OR as well as number of correctly classified cases. ORs of IL-1β, IL-6 and TNFα were 5.53, 1.14 and 1.27 (*p* < 0.001 for all) (Table 3).

### 3.5. Analysis of Relations between Inflammation-Driven Parameters in COPD Patients and Identification of COPD Clusters Regarding Systemic Inflammation

**Figure 2:**
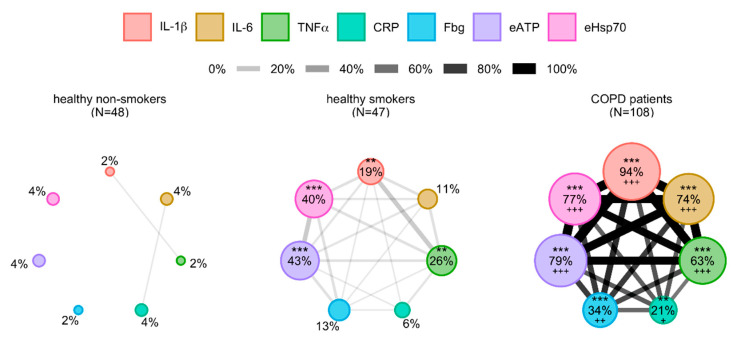
Network layout of cytokines combined with common inflammatory parameters and DAMPs in healthy non-smokers, healthy smokers and COPD patients. Parameters are shown as different nodes of the network whose size is in proportion with the prevalence of their abnormal values defined by 95th percentile of healthy non-smokers. Two nodes are linked when more than 1% of participants in the network share abnormal values of these two parameters, and width of a line is proportional to that proportion. DAMP–damage-associated molecular pattern; IL-1β—interleukin-1beta; IL-6—interleukin-6; TNFα—tumor necrosis factor alpha; CRP—C-reactive protein; Fbg—fibrinogen; eATP—extracellular adenosine-triphosphate; eHsp70—extracellular heat shock protein 70. ** *p* < 0.01, *** *p* < 0.001 in comparison to healthy non-smokers; +++ *p* < 0.001 in comparison to healthy smokers.

Based on the differences obtained in cytokines’ concentrations between healthy participants and COPD patients, cytokine network analysis and evaluation of predicting potential of investigated cytokines, IL-1β, IL-6 and TNFα showed statistically the most significant results. As systemic inflammation in COPD goes beyond increased production of cytokines, we wanted to broaden our view regarding complexity and networking of inflammatory parameters in blood of patients. Therefore, based on our previous research, common inflammatory parameters CRP and Fbg as well as DAMPs eATP and eHsp70 were included together with cytokines IL-1β, IL-6 and TNFα in further analysis. Now, we wanted to assess the relations between all those parameters, so additional network analysis was performed (Figure 2). Potential relations between them were also investigated in three groups of participants (healthy non-smokers, healthy smokers and COPD patients) with reference values of the 95th percentile of each parameter in healthy non-smokers as well (see Supplementary Table S2). Nodes were small in their size in healthy non-smokers, and only two links were present–one between CRP and IL-6 and the other between IL-1β and TNFα. Nodes with IL-1β (*p* < 0.01), TNFα (*p* < 0.01), eATP (*p* < 0.001) and eHsp70 (*p* < 0.001) were larger in healthy smokers in comparison to healthy non-smokers, and there were more links between all the parameters. Similar to cytokine network analysis, the most developed network could be seen in COPD patients where nodes of IL-1β, IL-6, TNFα, CRP, Fbg, eATP and eHsp70 were significantly larger in comparison to both healthy non-smokers (*p* < 0.001 for all except for CRP whose *p* value is <0.01) and healthy smokers (*p* < 0.001 for all except for CRP whose *p* value is <0.05, and Fbg whose *p* value is <0.01).

**Figure 3:**
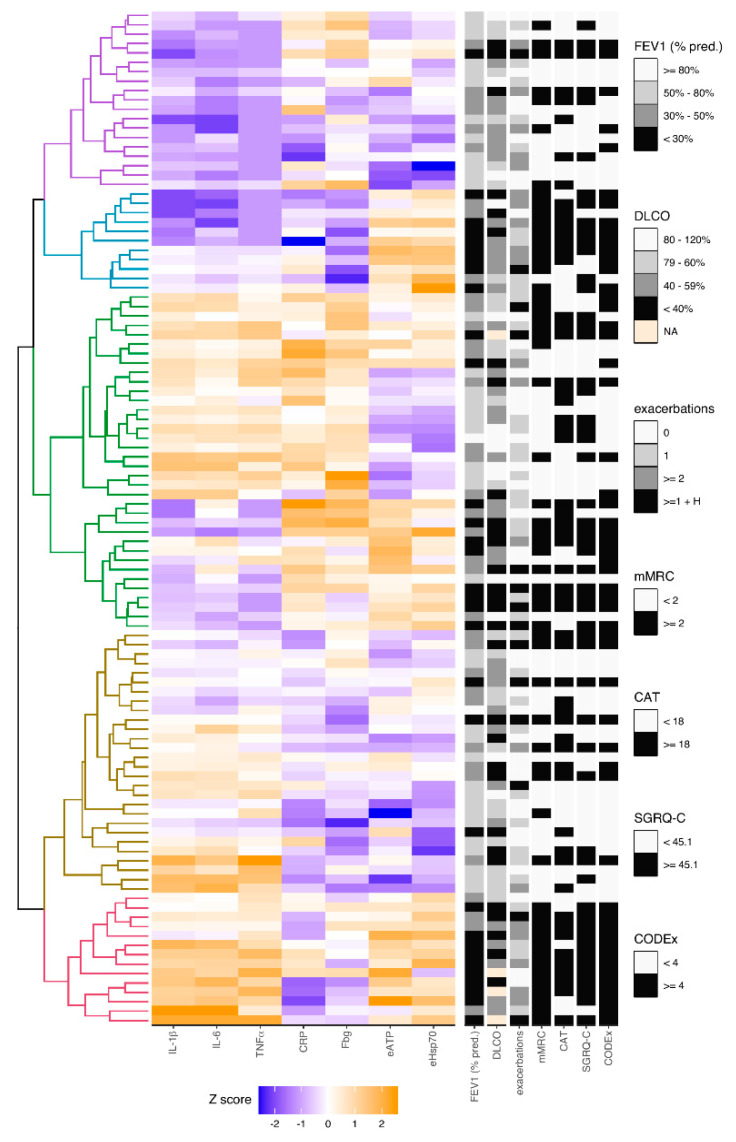
The heatmap of clusters of COPD patients regarding concentrations of cytokines (IL-1β, IL-6, TNFα), common inflammatory parameters (CRP, Fbg) and DAMPs (eATP, eHsp70). Hierarchical clustering analysis was used to characterize different phenotypes in COPD patients based on concentrations of cytokines (IL-1β, IL-6, TNFα), common inflammatory parameters (CRP, Fbg) and DAMPs (eATP, eHsp70). All concentrations were rank-based inverse normal transformed, and Euclidian correlation test with complete linkage was used for the clustering analysis. The heatmap shows colored squares, which present values of each parameter in each COPD patient. Five clusters of COPD patients were identified (left), and their clinical data (right) was included in the analysis, too. DAMP–damage-associated molecular pattern; IL-1β—interleukin-1beta; IL-6—interleukin-6; TNFα—tumor necrosis factor alpha; CRP—C-reactive protein; Fbg—fibrinogen; eATP—extracellular adenosine-triphosphate; eHsp70—extracellular heat shock protein 70; FEV_1_ (% pred.)—forced expiratory volume in one second (% predicted); DLCO—diffusing capacity for carbon monoxide; exacerbations–number of exacerbations reported in previous year; mMRC—modified Medical Research Council; CAT–COPD Assessment Test; SGRQ-C—St George’s Respiratory Questionnaire for COPD patients; CODEx—comorbidities, obstruction, dyspnoea, previous exacerbations; NA—not applicable; H–hospitalization.

In addition, hierarchical cluster analysis was conducted with seven variables and clinical data obtained from COPD patients (Figure 3, see Supplementary Table S3). FEV_1_ (% predicted) was included for the evaluation of airflow limitation-based severity. However, FEV_1_ is insufficient for the disease severity assessment, and there was a need for additional assessment by DLCO (as a measure of the diffusion properties of the alveolar capillary membrane), number of exacerbations, mMRC (for dyspnoea severity), CAT and SGRQ-C (for more comprehensive assessment of symptoms and quality of life related to health status) and multicomponent index CODEx that incorporates several variables with great emphasis on comorbidities. Division of COPD patients based on FEV_1_ was defined by GOLD guidelines [4], and there were four groups of COPD patients according to the severity of airflow limitation, as already described. Criteria for the diffusion limitation severity assessed by DLCO was defined by literature as well [30]. According to Fragoso et al., in our cluster analysis COPD patient that experienced two or more exacerbations or at least one exacerbation which led to hospitalization were considered to have a phenotype of exacerbator, while those that had less than two exacerbations during the previous year without hospitalization were considered to be non-exacerbators [31]. The division criteria for other parameters (mMRC, CAT, SGRQ-C, CODEx) were established by median values in COPD patients from the study.

Patients from cluster group 1 showed to have decreased concentrations of all three cytokines as well as mostly lower concentrations of eATP and eHsp70, while levels of CRP and Fbg seemed to be heterogeneous. Based on clinical data, those were predominantly the patients with mild clinical phenotype. COPD patients from cluster group 2 shared decreased values of all cytokines and they also mostly had lower levels of common inflammatory parameters, yet they had increased levels of DAMPs. All the patients from cluster group 2 were in GOLD 3 or GOLD 4 stages of the disease and were above the median of mMRC score. Most of them had a phenotype of exacerbator. Predominantly, patients in cluster group 2 had higher scores of CAT, SGRQ-C and CODEx. Cluster groups 3 and 4 showed to have various changes of all parameters included in the analysis, and there was no unambiguous clinical phenotype regarding observed changes. However, cluster group 4 showed to recruit more patients with milder clinical phenotype. Patients in cluster group 5 showed mostly lower values of CRP and Fbg and increased levels of all cytokines, eATP and eHsp70. All of them were in GOLD 3 and GOLD 4 stages of the disease, had at least one exacerbation in the previous year as well as lower DLCO (a few patients could not perform this analysis due to their severe symptoms; therefore, not applicable (NA) was designated for them on the heatmap). Almost whole group had great impact of dyspnoea on everyday life assessed by mMRC and great impact of comorbidities assessed by CODEx score, while most of the patients from the group had more severe symptoms that affect their quality of life.

### 3.6. Model Combined of IL-1β, eATP and eHsp70 as the Best Combination for Identifying COPD Patients

Finally, evaluation of predictive performances of additional parameters was performed by univariate logistic regression analysis, and it was shown that all parameters had potential in identifying COPD patients. CRP showed to have OR of 1.24 (95% CI = 1.09–1.41, *p* = 0.001), OR of Fbg was 2.55 (95% CI = 1.65–3.95, *p* < 0.001), while eATP showed to have OR of 20.16 (95% CI = 8.40–48.38, *p* < 0.001), and eHsp70 OR of 5.20 (95% CI = 3.02–8.96, *p* < 0.001). Based on all statistically significant results from univariate logistic regression analysis (cytokines in Table 3 as well as CRP, Fbg, eATP and eHsp70), multivariate logistic regression analysis suggested a model composed of IL-1β (OR = 3.58, 95% CI = 1.71–7.47), eATP (OR = 6.08, 95% CI = 1.79–20.64) and eHsp70 (OR = 3.10, 95% CI = 1.59–6.03). This model showed the greatest predictive performance in comparison to other models and successfully classified 91% of cases, while area under the curve (AUC) was 0.966 (95% CI = 0.931–0.987, *p* < 0.001) which was the highest one when compared to other AUCs of all investigated models.

## 4. Discussion

Our study showed that all investigated cytokines (IL-1α, IL-1β, IL-6, IL-8 and TNFα) were increased in plasma of COPD patients, yet there was no association with airflow obstruction and symptoms severity. Inflammation was more developed in healthy smokers when compared to healthy non-smokers, and even more in COPD patients which was assessed by two network analyses–one including only cytokines, and the other conducting IL-1β, IL-6 and TNFα as well as additional inflammation-associated parameters CRP, Fbg, eATP and eHsp70. Moreover, when all parameters and clinical data were included in cluster analysis, different COPD clusters were observed. Finally, our study suggested combination of IL-1β, eATP and eHsp70 as the best model in identifying COPD patients with 91% correctly classified cases.

COPD is characterized by persistent inflammation predominantly localized to the peripheral airways and lung parenchyma, but it is also recognized that systemic inflammation might have an important role in development and progression of the disease and its comorbidities. Underlying mechanism of systemic inflammation in COPD include oxidative stress and altered circulating levels of inflammatory mediators [6,7]. It was shown that IL-1α and IL-1β were increased in lung samples and sputum of COPD patients. Additionally, it has been established that IL-1β was increased in peripheral circulation, while no study, to the best of our knowledge, investigated levels of blood IL-1α in patients with COPD [32,33]. IL-6 was increased in blood samples as well as in samples obtained from respiratory system of COPD patients when compared to controls [2,12]. In addition, IL-8 was increased in plasma [7] and sputum [8] of COPD patients as well as TNFα [5,7]. Increased cytokine levels in patients with COPD from the current study in comparison to controls might be more related to the systemic inflammation present in stable COPD than to pulmonary function impairment. Cytokines are the markers of low-grade inflammation, which was significantly developed in COPD patients. This was observed from network analysis of all cytokines, and IL-1β, IL-6 and TNFα were the best discriminators of COPD patients with cytokine levels being >95th percentile of the group of healthy non-smokers. However, one should have in mind that those cytokines are not specific to COPD and that systemic inflammation develops later in the disease course. Therefore, association of cytokines with disease severity parameters and/or outcomes would be preferable. It was shown that IL-1β from peripheral circulation positively correlated with CRP and negatively with FEV_1_ [33]. Additionally, IL-1β is a dominant part of systemic pro-inflammatory response in COPD, and its high levels in sputum were associated with impaired lung function [11,34]. Negative correlation was also observed between IL-6 and FEV_1_ [35]. However, IL-6 was not associated with decline in lung function in COPD patients from the ECLIPSE cohort [11]. Cytokines IL-1β, IL-6 and TNFα showed to be associated with the severity of COPD [10,16,36,37]. Our study did not show an increase in cytokine concentration regarding the severity of airflow obstruction or symptoms severity and history of exacerbations. Association of cytokines with disease severity was not successfully replicated either in some other studies, which might indicate heterogeneity within patient populations [6,17,38]. Kleniewska et al. showed that COPD patients had increased IL-1β, IL-6 and TNFα in induced sputum, but there was no difference in their concentration in serum in comparison to healthy subjects. However, CRP and Fbg showed to be increased in serum of COPD patients from the same study [39]. Cigarette smoking is one of the main environmental contributors to the development and progression of COPD. Still, our results indicate that the elevation of plasma cytokine levels was a consequence of COPD rather than smoking status. Similar observation was present in the study by Selvarajah et al. [40]. Besides cytokines, other parameters significantly contribute to the inflammatory processes as well, so they should be also explored as potential diagnostic and/or therapeutic targets. Additionally, it is considered that spirometry, symptoms assessment and data regarding exacerbations are not sufficient to reflect entirely the heterogeneity of COPD [31], and similar levels of airflow obstruction might result with different outcomes depending on the presence or absence of persistent systemic inflammation [21]. Therefore, investigation of biomarkers is important because of the possible distinction of COPD patients based on various patterns in alteration of investigated biomarkers. In our previous publications that included the same subjects as the current study, common inflammatory parameters CRP and Fbg were increased in COPD patients, and their predictive potential was observed as well [22]. Additionally, in the same patients we also assessed eATP and eHsp70 and showed that both of those DAMPs were associated with smoking status, airflow obstruction severity as well as symptoms severity and history of exacerbations [23,24]. Moreover, their great predictive values and association with multicomponent clinical parameters used for COPD assessment indicate there might be eATP- and eHsp70-driven inflammation as a part of disease progression. When all aforementioned parameters were combined with IL-1β, IL-6 and TNFα in network analysis, it was shown that not all patients with stable COPD have increased systemic inflammatory parameters, and even already well-known inflammatory parameters like CRP and Fbg were increased in only 21% and 34% of COPD patients, respectively, in comparison to the 95th percentile of healthy non-smokers. This suggest that other parameters (IL-1β, IL-6, TNFα, eATP, eHsp70) might have more important role in ongoing inflammation. In addition, increasing compactness of connected lines indicates there is a significant progression of inflammation in COPD patients. Still, systemic inflammation does not have to be persistent. It was demonstrated after three years follow-up that if duration of systemic inflammation was at least one year, it could lead to worse COPD outcomes (all-cause mortality and/or exacerbation frequency) [21]. Therefore, our suggestion for the future studies is to include a measurement of the same parameters after prolonged period with the aim to assess the persistence of systemic inflammation. Furthermore, different phenotypes of COPD might be identified with the purpose of better prognosis, diagnosis, and targeted treatment of COPD. Some of advanced statistical techniques may prove to be useful in identifying candidate phenotypes. Cluster analysis encompasses different algorithms for grouping objects without a priori hypothesis. By applying cluster data analysis, we studied concentrations of various parameters and clinical data obtained from our patients with stable COPD. Therefore, the goal was to classify overall data into relatively homogeneous cluster groups. There were previous studies with cluster analyses of various cytokines in COPD. Cluster group of COPD patients with lower cytokines values regarding statin therapy was suggested as an important one by Marević et al. [41]. Additionally, comorbidity clusters of COPD patients were associated with systemic inflammation [42], while other studies suggested several COPD subtypes after applying cluster analyses that explored clinical variables and outcomes [43,44,45]. Our cluster analysis joined IL-1β, IL-6 and TNFα with CRP, Fbg, eATP and eHsp70 since they were recognized as potential parameters in identifying various subgroups among COPD patients with systemic inflammation. In addition, we included clinical data as referent variables. There were several observations from cluster analysis that are worth mentioning. Cluster groups 2 and 5 showed the worst status regarding all clinical variables in the study. Cluster group 2 had increased both eATP and eHsp70, while other investigated parameters were decreased. On the other hand, cluster group 5 encompassed patients with increased cytokines as well as eATP and eHsp70, while they had mostly intermediate or decreased levels of CRP and Fbg. Patients from both cluster groups were the ones with lowest FEV_1_ that was accompanied with lower health-related quality of life and possible significant impact of comorbidities assessed by CODEx. Considering that an increase in eATP and eHsp70 was accompanied by more severe clinical features, it could be suggested to include them in the assessment of COPD. Cluster groups 3 and 4 comprised most of the patients and it seems they represent the heterogeneity of COPD. All clinical variables significantly differed among these COPD patients. The heatmap shows there might be additional subgroups within groups 3 and 4 that were not separated by the cluster analysis. Finally, COPD patients from cluster group 1 had lower levels of all cytokines and mostly also of eATP and eHsp70, while CRP and Fbg differed. Predominantly, they showed to have mild to moderate clinical phenotype when the assessment of airflow limitation, exacerbations, symptoms severity and comorbidities were considered. Results from cluster analysis suggest there might be several patterns of inflammation in COPD patients, and similar was observed in the study of Rennard et al. [45]. It would be interesting to evaluate observed patterns in investigated parameters regarding commonly present comorbidities and potential effect of therapy. Cluster analysis might be a part of targeted approach towards future study designs, so the questions of interest could be directed to the specific COPD subgroups. Finally, when all statistically significant predictors from this study were included in multivariate logistic regression analysis, a combination of IL-1β, eATP and eHsp70 showed to have great performances in identifying COPD patients. The suggested model successfully classified 91% of all cases. Therefore, combined three-parameter model might have a great value in recognizing COPD patients, while patterns in concentrations of cytokines, CRP, Fbg, eATP and eHsp70 might be useful in identifying different COPD subgroups.

Several shortcomings are present in the current study. There were no patients in GOLD 1 stage or GOLD C group since COPD patients in GOLD 1 stage of the disease mostly do not contact their physician because of the very mild symptoms, while patients in GOLD C group usually do not manifest many symptoms and are not frequent exacerbators. However, larger number of participants in general should be considered in the further studies and a longitudinal study should be preferred over a cross-sectional case-control study.

## 5. Conclusions

Cytokines are one of the contributors in inflammatory processes present in COPD patients. However, by itself they are insufficient for the assessment of COPD, so additional biomarkers should be also evaluated. Models that include IL-1β, eATP and eHsp70 might prove to be useful in recognizing COPD patients because of its great predictive value, while combinations of IL-1β, IL-6 and TNFα with CRP, Fbg, eATP and eHsp70 might have a potential in differentiating COPD patients regarding clinical subgroups, with eATP and eHsp70 being particularly useful in identifying patients with severe COPD.

## Figures and Tables

**Table 1:** Demographic characteristics, spirometry parameters and cytokines’ concentrations in participants from the study.

	Total Healthy Subjects *n* = 95	Healthy Non-Smokers *n* = 48	COPD Patients *n* = 109	*p*_1_	*p*_2_
age	64 (46–83)	65 (52–83)	65 (45–87)	0.069	0.600
gender male female	49 46	23 25	69 40	0.121	0.104
FEV_1_ (L)	2.60 (2.12–3.19)	2.82 (2.28–3.19)	1.08 (0.69–1.60)	**<0.001**	**<0.001**
FEV_1_ (% pred.)	93.3 (86.4–104.2)	101.1 (90.6–110.4)	40.8 (27.9–61.7)	**<0.001**	**<0.001**
FVC (L)	3.35 (2.77–4.16)	3.58 (2.76–4.18)	2.28 (1.74–2.77)	**<0.001**	**<0.001**
FEV_1_/FVC (%)	80.6 (76.8–87.6)	83.0 (78.1–91.8)	51.3 (40.7–58.7)	**<0.001**	**<0.001**
IL-1α (pg/mL)	0.30 (0.30–0.97)	0.31 (0.31–0.71)	0.43 (0.30–2.13)	**0.003**	**0.007**
IL-1β (pg/mL)	0.10 (0.10–0.61)	0.10 (0.10–0.17)	6.90 (0.61–23.91)	**<0.001**	**<0.001**
IL-6 (pg/mL)	4.85 (3.45–7.09)	4.41 (3.29–6.17)	32.17 (10.64–64.30)	**<0.001**	**<0.001**
IL-8 (pg/mL)	6.36 (4.07–11.17)	6.22 (4.34–11.33)	8.73 (3.56–17.76)	**0.040**	**0.049**
TNFα (pg/mL)	0.40 (0.35–1.36)	0.35 (0.35–0.53)	8.24 (0.35–19.23)	**<0.001**	**<0.001**

Age was shown as median with minimum and maximum, while gender was presented as an absolute number. Results of spirometry and cytokines’ measurements were shown as median with interquartile range (IQR). Comparison of males and females was performed by Chi-squared test, while all other parameters were tested by Mann–Whitney Rank Sum test. Data were considered significant if *p* < 0.05. FEV_1_–forced expiratory volume in one second; FVC–forced vital capacity; IL-1α–interleukin-1alpha; IL-1β–interleukin-1beta; IL-6–interleukin-6; IL-8–interleukin-8; TNFα–tumor necrosis factor alpha. alpha; *p_1_*–statistical significance of differences between total healthy subjects and chronic obstructive pulmonary disease (COPD) patients; *p_2_*–statistical significance of differences between healthy non-smokers and COPD patients. All *p*-values that are <0.05 are in bold.

**Table 2:** Concentration of cytokines in healthy participants and COPD patients regarding the severity of airflow obstruction assessed by FEV_1_ (GOLD 2-4 stages) and the severity of symptoms and exacerbation history (GOLD A-D groups).

	IL-1α (pg/mL)	IL-1β (pg/mL)	IL-6 (pg/mL)	IL-8 (pg/mL)	TNFα (pg/mL)
controls *n* = 95	0.30 (0.30–0.97)	0.10 (0.10–0.61)	4.85 (3.45–7.09)	6.36 (4.07–11.17)	0.40 (0.35–1.36)
GOLD 2 *n* = 39	0.40 (0.30–2.37) ^1^	8.77 (0.70–20.40) ^1^	30.14 (10.54–58.01) ^1^	6.98 (3.27–15.25)	11.04 (0.39–19.37) ^1^
GOLD 3 *n* = 36	0.63 (0.30–2.04) ^1^	7.57 (0.75–22.63) ^1^	34.75 (8.25–56.75) ^1^	8.77 (3.50–23.59) ^1^	7.40 (0.77–14.08) ^1^
GOLD 4 *n* = 34	0.48 (0.30–1.60) ^1^	5.54 (0.56–42.23) ^1^	27.23 (12.51–106.87) ^1^	9.74 (4.56–22.89) ^1^	6.63 (0.35–31.37) ^1^
*p* *_1_*	**0.031**	**<0.001**	**<0.001**	**0.041**	**<0.001**
GOLD A *n* = 14	2.04 (0.30–3.09) ^1^	8.72 (3.55–20.63) ^1^	33.33 (11.95–56.65) ^1^	6.07 (3.55–14.00)	12.31 (3.34–18.65) ^1^
GOLD B *n* = 63	0.40 (0.30–1.84) ^1^	8.27 (0.56–25.78) ^1^	33.36 (10.85–71.16) ^1^	9.40 (3.64–19.89)	8.60 (0.35–19.46) ^1^
GOLD D *n* = 32	0.48 (0.30–2.56) ^1^	4.25 (0.53–21.72) ^1^	24.07 (8.25–52.93) ^1^	8.20 (3.37–18.60)	4.29 (0.35–17.28) ^1^
*p* *_2_*	**0.018**	**<0.001**	**<0.001**	**0.398**	**<0.001**

Data were presented as median with IQR after performing Kruskal–Wallis one-way analysis of variance test. Data were considered significant if *p* < 0.05. Afterwards, post-hoc analysis was performed. GOLD–Global Initiative for chronic obstructive pulmonary disease; IL-1α—interleukin-1alpha; IL-1β—interleukin-1beta; IL-6–interleukin-6; IL-8—interleukin-8; TNFα—tumor necrosis factor alpha; *p_1_*—statistical significance of differences between controls, GOLD 2, GOLD 3 and GOLD 4; *p_2_*—statistical significance of differences between controls, GOLD A, GOLD B and GOLD D. ^1^ statistically significant in comparison to controls. All *p*-values that are <0.05 are in bold.

**Table 3:** Univariate logistic regression analysis of all cytokines investigated.

	OR	*p*	95% CI	Cases Correctly Classified (%)
IL-1α	1.00	0.536	0.99–1.01	53
IL-1β	5.53	**<0.001**	2.05–14.90	84
IL-6	1.14	**<0.001**	1.08–1.19	80
IL-8	1.03	**0.010**	1.01–1.05	56
TNFα	1.27	**<0.001**	1.16–1.40	74

OR—odds ratio; CI—confidence interval; IL-1α—interleukin-1alpha; IL-1β—interleukin-1beta; IL-6—interleukin-6; IL-8—interleukin-8; TNFα—tumor necrosis factor alpha. All *p*-values that are <0.05 are in bold.

## Supplementary Materials

The following are available online at https://www.mdpi.com/2075-4418/10/12/1029/s1, Table S1: Levels of cytokines in all participants regarding their smoking status, Table S2: Values of 95th percentile of the parameters determined in healthy non-smokers that are used in network analyses, Table S3: Clinical characteristics and concentrations of cytokines (IL-1β, IL-6, TNFα), common inflammatory parameters (CRP, Fbg) and DAMPs (eATP, eHsp70) in COPD patients according to the five clusters after unsupervised hierarchical clustering analysis.
