# Supplementary material for: Combination of Systemic Inflammatory Biomarkers in Assessment of Chronic Obstructive Pulmonary Disease: Diagnostic Performance and Identification of Networks and Clusters
Source: Diagnostics (Basel). 2020 Nov 30;10(12):1029. doi: 10.3390/diagnostics10121029 (PMC7760570; doi:10.3390/diagnostics10121029)
Supplement: Supplementary file 1 [file diagnostics-10-01029-s001.pdf]

Combination of Systemic Inflammatory Biomarkers in Assessment of Chronic Obstructive Pulmonary Disease: Diagnostic Performance and Identification of Networks and Clusters

Iva Hlapčić [1], Daniela Belamarić [2], Martina Bosnar [2], Domagoj Kifer [3], Andrea Vukić Dugac [4,5] and Lada Rumora [1*]

[1] University of Zagreb, Faculty of Pharmacy and Biochemistry, Department of Medical Biochemistry and Haematology, Zagreb, Croatia

[2] Fidelta Ltd., Zagreb, Croatia

[3] University of Zagreb, Faculty of Pharmacy and Biochemistry, Department of Biophysics, Zagreb, Croatia

[4] University Hospital Centre Zagreb, Clinical Department for Lung Diseases Jordanovac, Zagreb, Croatia

[5] University of Zagreb, School of Medicine, Zagreb, Croatia

* Correspondence: lada.rumora@pharma.unizg.hr; Tel.: +38516394782, Fax: +38514612716

Table 1. Levels of cytokines in all participants regarding their smoking status.

	healthy non-smokers N = 48	healthy smokers N = 47	COPD non-smokers N = 5	COPD former smokers N = 75	COPD smokers N = 29	P
IL-1α (pg/ml)	0.30 (0.30 - 0.71)	0.30 (0.30 - 1.78)	0.30 (0.30 - 1.43)	0.40 (0.30 - 1.84)	1.57 (0.30 - 2.66) [1,2]	**0.010**
IL-1β (pg/ml)	0.10 (0.10 - 0.16)	0.10 (0.10 - 2.76)	0.62 (0.28 - 2.24) [1,2]	8.24 (0.56 - 26.23) [1,2]	9.65 (1.52 - 17.54) [1,2]	**<0.001**
IL-6 (pg/ml)	4.41 (3.29 - 6.17)	5.37 (3.79 - 8.01)	10.40 (2.88 - 15.51)	36.05 (10.75 - 72.66) [1,2,3]	33.05 (11.09 - 52.03) [1,2,3]	**<0.001**
IL-8 (pg/ml)	6.22 (4.34 - 11.33)	6.36 (3.99 - 11.17)	3.25 (2.32 - 6.92)	8.20 (3.61 - 20.22)	9.65 (4.39 - 17.54)	0.172
TNFα (pg/ml)	0.35 (0.35 - 0.53)	0.70 (0.35 - 3.04) [1]	0.35 (0.35 - 0.71)	8.18 (0.46 - 23.32) [1,2,3]	11.08 (1.13 - 17.55) [1,2,3]	**<0.001**

Data were tested by Kruskal-Wallis one-way analysis of variance and presented as median with IQR. Results were statistically significant if $P<0.05$. Afterwards, post-hoc analysis was performed.

IL-1α – interleukin-1alpha; IL-1β – interleukin-1beta; IL-6 – interleukin-6; IL-8 – interleukin-8; TNFα – tumour necrosis factor alpha.

[1] statistically significant in comparison to healthy non-smokers;

[2] statistically significant in comparison to healthy smokers;

[3] statistically significant in comparison to COPD non-smokers.

Table 2. Values of 95th percentile of the parameters determined in healthy non-smokers that are used in network analyses.

network analysis of all cytokines	
parameter	95th percentile value
IL-1α (pg/ml)	6.18
IL-1β (pg/ml)	0.10
IL-6 (pg/ml)	10.48
IL-8 (pg/ml)	26.14
TNFα (pg/ml)	2.65
network analysis of selected parameters	
parameter	95th percentile value
IL-1β (pg/ml)	0.10
IL-6 (pg/ml)	10.48
TNFα (pg/ml)	2.65
CRP (mg/l)	5.15
Fbg (g/l)	4.2
eATP (μmol/l)	1.10
eHsp70 (ng/ml)	0.74

Table S3. Clinical characteristics and concentrations of cytokines (IL-1β, IL-6, TNFα), common inflammatory parameters (CRP, Fbg) and DAMPs (eATP, eHsp70) in COPD patients according to the five clusters after unsupervised hierarchical clustering analysis.

parameter	CLUSTER 1<br>N=19	CLUSTER 2<br>N=11	CLUSTER 3<br>N=36	CLUSTER 4<br>N=28	CLUSTER 5<br>N=14	P	after adjusted P-value *
FEV_1<br>(% pred.)	61.8<br>(40.4 – 61.8)	26.0<br>(20.8 – 30.0)	43.8<br>(29.4 – 56.9)	54.5<br>(34.4 – 64.0)	27.3<br>(23.1 – 30.6)	**<0.001**	1 vs. 2<br>1 vs. 3<br>1 vs. 5<br>2 vs. 3<br>2 vs. 4<br>3 vs. 5<br>4 vs. 5
DLCO	55.7<br>(45.1 -74.4)	44.6<br>(37.5 – 46.2)	50.1<br>(36.3 – 74.2)	56.7<br>(36.2 – 70.0)	40.7<br>(34.8 – 44.6)	0.319	NS
exacerbations (N)<br>0<br>1<br>≥2<br>≥1 + H	 8<br>5<br>5<br>1	 1<br>4<br>5<br>1	 13<br>14<br>3<br>6	 13<br>9<br>2<br>4	 3<br>4<br>5<br>2	0.130	NS
mMRC	1<br>(1 – 2)	3<br>(2 – 3)	1<br>(1 - 2)	1<br>(1 – 2)	2<br>(2 - 3)	**<0.001**	1 vs. 2<br>1 vs. 5<br>2 vs. 3<br>2 vs. 4<br>3 vs. 5<br>4 vs. 5
CAT	15<br>(11 - 20)	20<br>(16 – 27)	17<br>(13 – 24)	17<br>(8 – 21)	22<br>(14 – 26)	**0.016**	NS
SGRQ-C	32.5<br>(24.1 – 49.8)	59.5<br>(45.5 – 79.4)	47.3<br>(31.3 – 62.5)	35.2<br>(20.5 – 47.1)	64.2<br>(54.7 – 69.0)	**<0.001**	1 vs. 2<br>1 vs. 5<br>2 vs. 4<br>3 vs. 4<br>4 vs. 5

CODEx	1<br>(1 – 3)	6<br>(5 – 6)	4<br>(2 – 5)	2<br>(1 – 4)	5<br>(4 - 6)	**<0.001**	1 vs. 2<br>1 vs. 5<br>2 vs. 3<br>2 vs. 4<br>3 vs. 5<br>4 vs. 5
IL-1β<br>(pg/ml)	0.38<br>(0.24 – 0.65)	0.21<br>(0.12 – 3.82)	13.22<br>(1.08 – 25.90)	8.81<br>(3.90 – 20.25)	47.55<br>(12.98 – 56.40)	**<0.001**	1 vs. 3<br>1 vs. 4<br>1 vs. 5<br>2 vs. 3<br>2 vs. 4<br>2 vs. 5<br>3 vs. 5<br>4 vs. 5
IL- 6<br>(pg/ml)	3.84<br>(1.87 – 9.26)	10.72<br>(1.56 – 14.73)	39.91<br>(29.98 – 72.15)	37.67<br>(19.08 – 63.71)	118.73<br>(40.21 – 189.82)	**<0.001**	1 vs. 3<br>1 vs. 4<br>1 vs. 5<br>2 vs. 3<br>2 vs. 4<br>2 vs. 5<br>3 vs. 5<br>4 vs. 5
TNFα<br>(pg/ml)	0.35<br>(0.35 – 0.35)	0.35<br>(0.35 – 4.22)	12.31<br>(0.77 – 20.00)	9.22<br>(5.77 – 18.12)	27.56<br>(18.48 – 56.77)	**<0.001**	1 vs. 3<br>1 vs. 4<br>1 vs. 5<br>2 vs. 3<br>2 vs. 4<br>2 vs. 5<br>3 vs. 5<br>4 vs. 5
CRP<br>(mg/l)	2.21<br>(1.09 – 4.53)	1.75<br>(0.98 – 2.96)	5.34<br>(3.82 – 8.35)	1.24<br>(0.89 – 1.77)	1.21<br>(0.77 – 3.21)	**<0.001**	1 vs. 3<br>2 vs. 3<br>3 vs. 4

							3 vs. 5
Fbg (g/l)	3.8 (3.6 – 4.5)	3.0 (2.5 – 3.3)	4.5 (4.2– 5.1)	3.5 (3.1 – 3.7)	3.7 (3.5 – 3.9)	**<0.001**	1 vs. 2<br>1 vs. 3<br>1 vs. 4<br>2 vs. 3<br>2 vs. 4<br>2 vs. 5<br>3 vs. 4<br>3 vs. 5
eATP (μmol/l)	1.19 (0.93 – 1.52)	1.91 (1.84 – 2.23)	1.63 (1.24 – 2.04)	1.38 (1.12 – 1.52)	1.94 (1.92 – 2.33)	**<0.001**	1 vs. 2<br>1 vs. 3<br>1 vs. 5<br>2 vs. 4<br>3 vs. 4<br>3 vs. 5<br>4 vs. 5
eHsp70 (ng/ml)	0.99 (0.58 – 1.49)	3.41 (2.94 – 6.84)	2.06 (1.09 – 2.96)	0.82 (0.51 – 1.54)	3.45 (3.15 – 5.10)	**<0.001**	1 vs. 2<br>1 vs. 3<br>1 vs. 5<br>2 vs. 3<br>2 vs. 4<br>3 vs. 4<br>3 vs. 5<br>4 vs. 5

DAMP – damage-associated molecular pattern; FEV_1– forced expiratory volume in one second; DLCO – diffusing capacity for carbon monoxide; exacerbations – number of exacerbations reported in previous year; mMRC – modified Medical Research Council; CAT – COPD Assessment Test; SGRQ-C – St George's Respiratory Questionnaire for COPD patients; CODEx – comorbidities, obstruction, dyspnoea, previous exacerbations; IL-1β – interleukin-1beta; IL-6 – interleukin-6; TNFα – tumour necrosis factor alpha; CRP – C-reactive protein; Fbg – fibrinogen; eATP – extracellular adenosine-triphosphate; eHsp70 – extracellular heat shock protein 70; H – hospitalization; NS – not significant after the adjustment of P-value.

* significant differences between the groups after the adjustment of P-value by Benjamini-Hochberg method.
